# A narrative review on sacubitril/valsartan and ventricular arrhythmias

**DOI:** 10.1097/MD.0000000000029456

**Published:** 2022-07-08

**Authors:** Zhaoyang Wei, Meiwei Zhang, Qian Zhang, Linan Gong, Xiangyu Wang, Zanzan Wang, Ming Gao, Zhiguo Zhang

**Affiliations:** a Department of Cardiology, the First Hospital of Jilin University, Changchun, Jilin Province, China.

**Keywords:** angiotensin receptor neprilysin inhibitor, antiarrhythmic effect, LCZ696, myocardial fibrosis, sacubitril/valsartan, ventricular arrhythmias

## Abstract

Sacubitril/valsartan, the first angiotensin receptor neprilysin inhibitor approved by the Food and Drug Administration for marketing, has been shown to reduce the risk of cardiovascular death or heart failure hospitalization and improve symptoms in patients with chronic heart failure with a reduced ejection fraction. However, some researchers have also found that sacubitril/valsartan has an antiarrhythmic effect. The mechanism by which sacubitril/valsartan reduces the mortality associated with malignant ventricular arrhythmias is not precise. Many studies have concluded that ventricular arrhythmia is associated with a reduction in myocardial fibrosis. This article reviews the current understanding of the effects of sacubitril/valsartan on the reduction of ventricular arrhythmia and explains its possible mechanisms. The results of this study suggest that sacubitril/valsartan reduces the occurrence of appropriate implantable cardioverter-defibrillator shocks. Meanwhile, sacubitril/valsartan may reduce the occurrence of ventricular arrhythmias by affecting 3 pathways of B-type natriuretic peptide, Angiotensin II, and Bradykinin. The conclusion of this study is that sacubitril/valsartan reduces the number of implantable cardioverter-defibrillator shocks and ventricular arrhythmias in heart failure with reduced ejection fraction patients.

## 1. Introduction

In recent years, angiotensin receptor neprilysin inhibitor (ARNI) has gradually replaced angiotensin-converting enzyme inhibitors (ACEI) to become the cornerstone of treatment for heart failure and a reduced ejection fraction since ARNI was better at reducing the risk of cardiac death. Sacubitril/valsartan, the first ARNI approved by the Food and Drug Administration, is mainly composed of sacubitril and valsartan in the form of a sodium salt complex in a 1:1 ratio.^[1]^ Research into the use of sacubitril/valsartan for treating heart failure has made substantial progress, including positive results from many large-scale clinical trials, such as the PARADIGM-HF trial (Prospective Comparison of ARNI With ACEI to Determine Impact on Global Mortality and Morbidity in Heart Failure Trial)^[2,3]^ and the PARAMOUNT trial (Prospective comparison of ARNI with ARB [angiotensin receptor blocker] on Management Of heart failUre with preserved ejectioN fracTion trial).^[4]^ However, with the widespread use of sacubitril/valsartan, some researchers have found that sacubitril/valsartan reduced the incidence of ventricular arrhythmia in patients. This leads to the question of whether sacubitril/valsartan has the value of clinical application as an antiarrhythmic drug.^[5]^ This review supplements the previous review and aims to evaluate the potential antiarrhythmic uses of sacubitril/valsartan and elucidate their possible mechanisms.

## 2. What was found in previous research

Sacubitril/valsartan is mainly used in patients with chronic heart failure with a reduced ejection fraction (HFrEF). Therefore, current research on the antiarrhythmic effect of sacubitril/valsartan has focused on this patient population. In 2014, the PARADIGM-HF trial concluded that sacubitril/valsartan was superior to enalapril in reducing the risks of death and hospitalization for heart failure, but the specific mechanism was not fully elucidated at that time. Compared with enalapril therapy, sacubitril/valsartan therapy led to improved left ventricular ejection fraction (LVEF), reduced ventricular arrhythmia inducibility, and upregulated the expression of K + channel proteins.^[6]^ Desai et al^[7]^ think the reduction in both sudden cardiac deaths and deaths from worsening heart failure was probably due to the modification of the substrate for fatal ventricular arrhythmias such as myocardial fibrosis, ventricular hypertrophy, and progressive ventricular remodeling. However, no clinical trials have been conducted to verify these hypotheses. Until 2018, prospective research found that the application of sacubitril/valsartan decreased ventricular arrhythmias and appropriate implantable cardioverter-defibrillator (ICD) shocks in HFrEF patients under home monitoring compared with angiotensin inhibition.^[8]^ Subsequently, some scholars proposed the hypothesis of sacubitril/valsartan as an antiarrhythmic drug and believed that applying sacubitril/valsartan in combination with other drugs to treat heart failure would narrow the additional survival benefit provided by an ICD.^[5]^ Researchers believe that the need for cardiac implantable devices (e.g., ICD) in patients with HFrEF could be reduced significantly by treatment with sacubitril/valsartan.^[9]^ Dominguez et al^[10]^ think that the improvement in left ventricular reverse remodeling and ejection fraction after the use of sacubitril/valsartan decreased the need for ICD implantation. However, other researchers believe that clinical decision making cannot merely be based on echocardiographic results. The evaluation of a patient’s risk of arrhythmia should be the first choice.^[11]^

**Figure 1.**
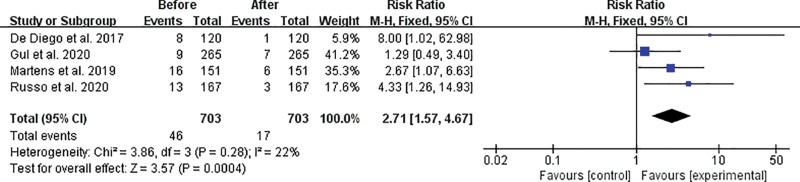
Data analysis of the number of appropriate ICD shocks before and after taking sacubitril/valsartan. There are significant differences between the 2 groups in appropriate ICD shocks was observed, but with mild heterogeneity. CI = confidence interval, ICD = implantable cardioverter-defibrillator. Favors [control] = before taking sacubitril/valsartan. Favors [experimental] = after taking sacubitril/valsartan.

Regarding whether the initiation of sacubitril/valsartan is associated with fewer ICD interventions, we selected 4 studies for data analysis (Methodology for selecting studies for data analysis can be found in part 3), and the results are presented in a forest plot (Fig. 1). As can be seen from Figure 1, sacubitril/valsartan reduces the occurrence of appropriate ICD shocks (95% confidence interval [CI] [1.57,4.67], Z = 3.57 [*P* < .001]).^[8,12–14]^ Similarly, a 6-minute walk test and echocardiographic evaluation of patients who underwent both ICD implantation and sacubitril/valsartan showed that even in ICD patients with a higher risk of arrhythmia, clinical, and functional improvements were significantly improved.^[15]^

On the contrary, Vicent et al^[16]^ report 6 cases of arrhythmic storm shortly after the initiation of sacubitril/valsartan, but the outcome is not enough to infer a cause-and-effect relationship between taking sacubitril/valsartan and arrhythmia due to the small sample size. Soon after, other researchers also reported cases of ventricular arrhythmia during the application of sacubitril/valsartan.^[17]^ Similarly, in 2019, a retrospective study found that sacubitril/valsartan did not reduce the risk of ventricular arrhythmias in chronic HFrEF over 1 year of follow-up.^[18]^ Somberg and Molnar^[19]^ believed that the patients included in the experiment of Vicent et al were very sick patients, all of whom were more likely to develop arrhythmia, the arrhythmia storm after drug initiation, and could have been a coincidence. Additionally, a study by Gatti et al^[20]^ showed that sudden cardiac death occurred early after sacubitril/valsartan administration. While Vincent et al^[21]^ believe that the antiarrhythmic effect of sacubitril/valsartan is unquestionable, an initial proarrhythmic effect in a subgroup of patients could still occur. At the same time, more patients who develop ventricular arrhythmia after sacubitril/valsartan administration have been reported. Discontinuing sacubitril/valsartan and switching to essential and conventional medications may better control ventricular arrhythmia in some patients.^[22]^

Prospective research evaluated chronic heart failure patients with LVEF ≤ 40% by comparing electrocardiographic parameters and the mechanical dispersion index before and after sacubitril/valsartan therapy. In conclusion, sacubitril/valsartan therapy has a reductive effect on the QTc interval, QRS duration, and mechanical dispersion index.^[23]^ In addition, de Carlos et al^[8]^ showed that sacubitril/valsartan reduced the incidence of sudden cardiac death caused by ventricular tachycardia. The mechanisms underlying this effect include recovery of contractile function, reduced fibrosis, reduced wall stress, and reconstruction of ion channels.

Sacubitril/valsartan improves LVEF and significantly reduces emergency treatment rate in patients with HFrEF.^[24]^ Patients who received a higher initial dose of sacubitril/valsartan tended to have a better recovery of left ventricular function. In patients whose left ventricular function has recovered, a gradual decrease in the dose of sacubitril/valsartan is associated with deterioration of restored cardiac function.^[25]^ The results of the PARADIGM-HF trial showed that LVEF was an effective predictor of prognosis in patients with heart failure, and that the use of sacubitril/valsartan significantly reduced cardiovascular mortality and the number of hospitalizations due to heart failure.^[26]^ Sudden cardiac death in patients with heart failure is primarily associated with the occurrence of ventricular arrhythmia. Although the current research results show that sacubitril/valsartan can reduce the occurrence of ventricular arrhythmia, more evidence is needed to support the clinical use of sacubitril/valsartan as an antiarrhythmic drug.

## 3. Methodology for selecting literature for data analysis

### 3.1. Search strategy

Studies were identified by searching electronic databases (Emabase and PubMed) from their inception to January 20, 2022. Our search query in Pubmed is (((“sacubitril and valsartan sodium hydrate drug combination”[All Fields] OR Sacubitril valsartan[Text Word] OR LCZ696[Text Word] OR Entresto[Text Word] OR AHU377[Text Word] OR ARNI[Text Word] OR Angiotensin Receptor-Neprilysin Inhibit*[Text Word])) AND ((“heart ventricles”[MeSH Terms] OR ventricular[Text Word]))) AND (((ICD[All Fields] OR “defibrillators, implantable”[MeSH Terms] OR Implantable cardioverter-defibrillator[Text Word]) OR (“arrhythmias, cardiac”[MeSH Terms] OR arrhythmias[Text Word] OR (Dysrhythmia*[Text Word] AND Cardiac[Text Word]) OR Arrythmia[Text Word]) OR (“death, sudden, cardiac”[MeSH Terms] OR sudden cardiac death[Text Word] OR Sudden Cardiac Arrest[Text Word]))). No language restriction was applied. The reference lists of identified articles were also reviewed to identify additional citations.

### 3.2. Eligibility criteria

Studies with the following characteristics were considered eligible: included patients are taking sacubitril/valsartan; using implantable cardioverter-defibrillator (ICD) to monitor the incidence of arrhythmias and ICD shocks in patients taking sacubitril/valsartan; and the number of ICD shocks before and after taking sacubitril/valsartan was compared. Case reports, case series, conference presentations, reviews, and expert opinions were excluded from our analysis.

### 3.3. Data extractions and quality appraisal

**Figure 2.**
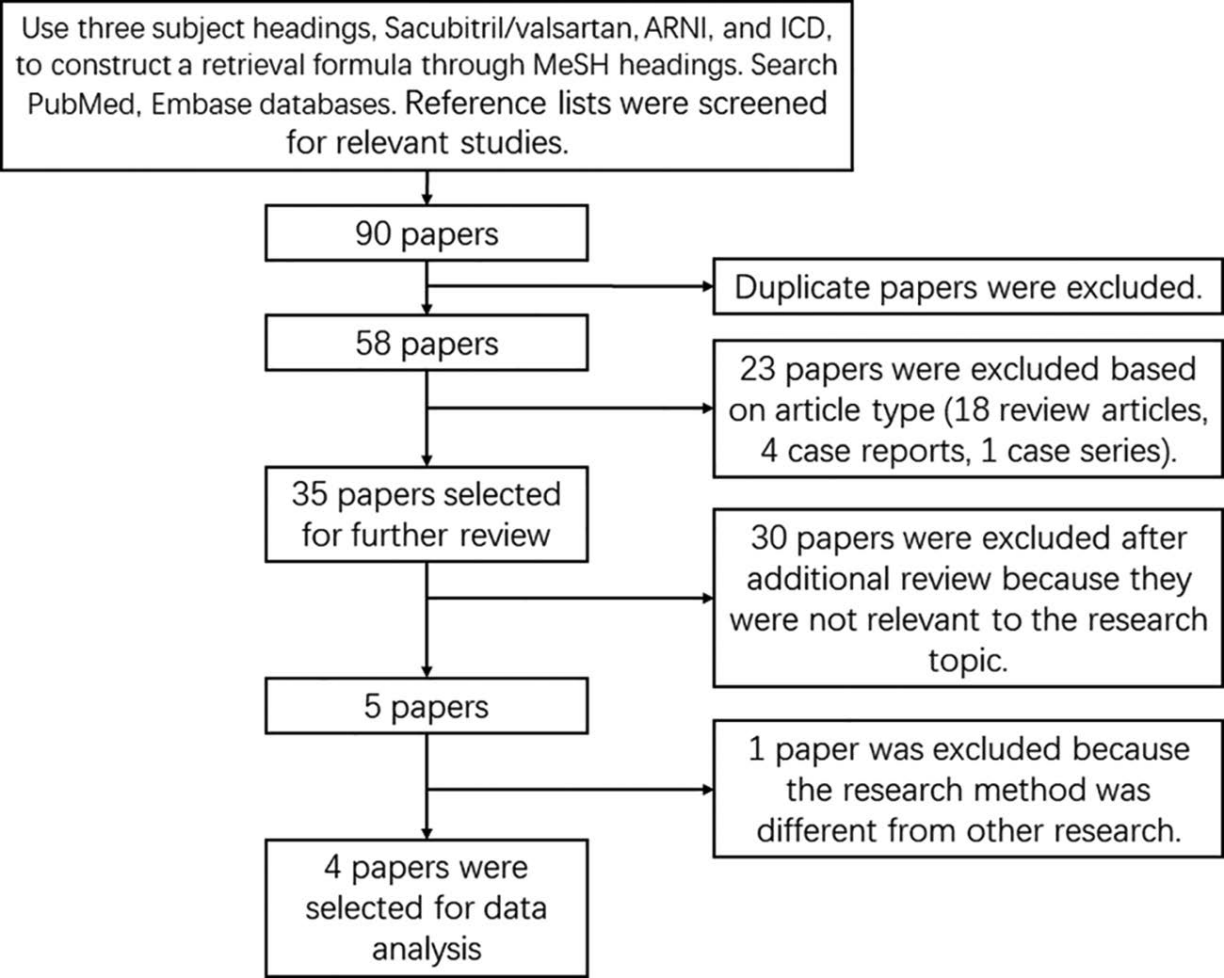
Methods of selecting articles for inclusion in data analysis. ARNI = angiotensin receptor neprilysin inhibitor, ICD = implantable cardioverter-defibrillator, MeSH = Medical Subject Headings.

Two investigators (Z.W. and M.Z.) independently screened all titles, abstracts and manually searched the full text of all relevant studies that met the inclusion criteria. Included articles were independently reviewed by each investigator, and disagreements were resolved by discussion. A total of 90 studies were identified using specified search criteria. After a detailed evaluation of these studies, 4 relevant studies that met our predefined eligibility criteria were included (Fig. 2).

### 3.4. Statistical analysis

Data were analyzed using RevMan 5.4 software. I^2^ was used to test for heterogeneity between studies and I^2^ ≤ 25% was assumed to indicate homogeneity. Results that met these criteria were then included in the fixed-effects model.

## 4. Possible mechanisms underlying the antiarrhythmic effect of sacubitril/valsartan

The effect of sacubitril/valsartan in reducing ventricular arrhythmia involves neprilysin and renin-angiotensin-aldosterone system (RAAS) inhibition. Both involve many pathways. Therefore, the exact mechanism of the effect of sacubitril/valsartan in reducing ventricular arrhythmia is very complicated. Previous research has shown that the initiation of sacubitril/valsartan administration could reduce ventricular arrhythmia.^[8,12–14]^ Similarly, the conclusion of the PARADIGM-HF trial showed that sacubitril/valsartan reduces the patient’s risk of death. However, the mechanism underlying the reduction of ventricular arrhythmia has not been fully elucidated. A study investigating the effect of sacubitril/valsartan on the occurrence of ventricular arrhythmia in a rat model of myocardial infarction heart failure found that the recovery of myocardial fibrosis may explain the reduced ventricular arrhythmia inducibility, which may partly explain the mechanisms of decreased sudden cardiac death in the PARADIGM-HF trial.^[27]^ Another study showed that ARNI ameliorates postinfarction ventricular arrhythmias in rats with ischemic cardiomyopathy through sympathetic nerve remodeling and structural remodeling.^[28]^ In a study on rabbits, sacubitril/valsartan reduced fibrosis and the expression of phosphorylated calcium/calmodulin-dependent protein kinase II protein.^[29]^ Related to this is that some fibrosis formations are Ca2+-dependent.^[30]^ Previous studies have shown that sacubitril/valsartan reduces ventricular arrhythmias by changing the ion concentration and ion channels.^[31]^ Here, we describe the mechanism by which sacubitril/valsartan reduces arrhythmia by reducing myocardial fibrosis.

On the one hand, ventricular arrhythmia can be induced by impulse initiation and impulse propagation abnormalities, including many mechanisms.^[32]^ Many of these mechanisms are associated with the structural and molecular changes in cardiomyocytes, including myocardial fibrosis is one of them. Various studies have shown that fibrosis plays an essential role in cardiac arrhythmias.^[33,34]^ Furthermore, improving myocardial fibrosis with sacubitril/valsartan may reduce the occurrence of ventricular arrhythmias.^[35]^ On the other hand, after entering the human body, sacubitril/valsartan is decomposed into sacubitril and valsartan, which are further metabolized into LBQ657 to play a role in inhibition of neprilysin.^[1]^ However, >50 putative peptide substrates of neprilysin have been proposed until 2016. Many of these proteins have unclear functions. The substrates of cardiorenal neprilysin include natriuretic peptides, angiotensin, endothelin, adrenomedullin, and bradykinin.^[36]^ Sacubitril/valsartan works by inhibiting the function of neprilysin by reducing the degradation of these substances. Moreover, valsartan inhibits angiotensin type 1 (AT1) receptor.^[37]^ In general, sacubitril and valsartan synergistically inhibit myocardial fibrosis and ventricular arrhythmia via different mechanisms.

Accordingly, the majority of studies on the mechanism of ventricular arrhythmia reduction while taking sacubitril/valsartan are based on the changes in the bioactive peptides mentioned above. Among these 5 substrates, endothelin and adrenomedullin increase ventricular arrhythmia.^[38,39]^ Therefore, the mechanism by which sacubitril/valsartan reduces ventricular arrhythmia is described in 3 sections.

### 4.1. B-type natriuretic peptide pathway

Three main natriuretic peptides are produced by the human body: atrial natriuretic peptide, B-type natriuretic peptide (BNP), and C-type natriuretic peptide. Atrial natriuretic peptide and C-type natriuretic peptide have a short half-life, and BNP plays a significant role in regulating cardiovascular physiology.^[40]^ BNP is released mainly by ventricular myocytes in precursor hormone form, which is then cleaved into 2 parts: BNP and N-terminal pro-BNP. The main effects of BNP are diuresis and natriuretic and vasodilator effects.^[41]^

BNP is a well-recognized predictor of sudden cardiac death in heart failure patients with impaired LVEF or heart failure with ischemic disease because it is directly related to the progressive deterioration of heart failure.^[42,43]^ Some studies have shown that a decrease in myocardial fibrosis and electrophysiological remodeling reduces the occurrence of ventricular arrhythmia.^[44,45]^ At the same time, injection of exogenous BNP also showed that recombinant human BNP improved cardiac function, increased ejection fraction, dilated vessels, and had antifibrotic effects.^[46]^ In addition, a meta-analysis defining the predictors of sudden cardiac death and ventricular arrhythmia in patients with ICD found that high baseline BNP or N-terminal pro-BNP levels were independently associated with the occurrence of ventricular tachyarrhythmia.^[47]^

Gene deletion experiments in mice have indicated that BNP preventing cardiac fibrosis.^[48]^ Current research suggests that the antifibrotic effect of BNP is related to guanylate cyclase. After binding to the corresponding receptors, BNP catalyzes the conversion of guanosine triphosphate to guanosine cyclophosphate by activating guanylate cyclase. After guanylate cyclase activation, cyclic guanosine monophosphate is produced in large quantities. Protein kinase G (PKG) is activated to act on the effector protein phospholamban and protein kinase C, increasing sarcoplasmic reticulum calcium uptake through phospholamban phosphorylation, and has a synergistic effect with valsartan in inhibiting protein kinase C channels, which ultimately leads to the reduction of cardiac fibrosis. However, its specific mechanism of action in the nucleus remains unclear.^[36,49–52]^

### 4.2. Angiotensin II pathway

Long-term myocardial fibrosis and cardiac remodeling can increase the occurrence of malignant arrhythmias.^[53]^ After entering the human body, valsartan inhibits the actions of angiotensin II (Ang II) by antagonizing the AT1 receptor, thus reducing blood pressure, reversing cardiac remodeling, and playing a therapeutic role in heart failure.^[54]^ The aforementioned studies have shown that RAAS activation promotes ventricular fibrosis. These findings suggest a direct interaction between the RAAS and cardiac fibroblasts. Myocardial fibrosis can be prevented by inhibiting RAAS.

A previous study found that Ang II could stimulate collagen synthesis and inhibit matrix metalloproteinase I activity, thus leading to excessive collagen accumulation and causing structural changes in myopathy.^[55]^ Metalloproteinase I is a critical enzyme that degrades the fibrous collagen in the interstitium of the heart. Its inhibition leads to the excessive accumulation of collagen, which in turn leads to myocardial fibrosis. Similarly, in cultured adult cardiac fibroblasts, Ang II and aldosterone have been shown to stimulate collagen synthesis and Ang II inhibits the activity of metalloproteinase I.^[55]^

AT1 receptor binding of Ang II also activates tyrosine kinase phosphorylation and phospholipase C, leading to the activation of downstream signals, such as mitogen-activated protein kinase, ultimately leading to other cellular effects of gene transcription changes. Ang II activation of the AT1 receptor leads to the opening of Ca2 + channels and extracellular Ca2 + into cells.^[56]^ Ang II-induced AT1 receptor activation stimulates a variety of intracellular signaling pathways through G proteins, including phospholipase C, phospholipase D, phospholipase A2, Ca2 + channels, adenylate cyclase (AC), and mitogen-activated protein kinase.^[57]^

After Ang II acts on cardiomyocytes, the AT1 receptor in the cardiomyocytes is activated, and then the signal is transmitted to phospholipase C through the G protein. Phospholipase C transfers 4,5-bisphosphate phosphatidylinositol, which is hydrolyzed into inositol triphosphate and diacylglycerol. Then, diacylglycerol activates protein kinase C, which acts on the nucleus to produce biological effects. Simultaneously, inositol triphosphate acts on the nuclear inositol triphosphate receptor, causing the intracellular Ca2 + concentration to increase. Intracellular Ca2 + and calmodulin combine to form the calcium/calmodulin-dependent protein kinase II. Then, calcium/calmodulin-dependent protein kinase II acts on the effector protein to produce biological effects.^[58,59]^ More importantly, the combination of sacubitril and valsartan synergistically prevented myocardial cell death, showing that valsartan enhanced the effect of sacubitril.^[45]^

### 4.3. Bradykinin pathway

Neprilysin degrades several peptides, including bradykinin.^[60]^ Bradykinin is a substrate of both angiotensin-converting enzymes and neprilysin.^[61]^ Studies have suggested that neprilysin seems to be more critical than the angiotensin-converting enzyme in removing bradykinin from cardiac fibroblasts and cardiomyocytes.^[62]^ There is some evidence that bradykinin may play a role in the therapeutic benefits of sacubitril/valsartan therapy and angioedema associated with this therapy.^[63]^ Furthermore, studies have shown that bradykinin promotes vasodilation, prevents ischemia and fibrosis, and highlights its cardioprotective effects. These effects may be related to the reduction in ventricular arrhythmia.^[31,64]^ Bradykinin is a nonapeptide produced by the cleavage of high-molecular-weight kininogen after factor XII (FXII) and prekallikrein (PK) in plasma are activated to form FXIIa and PKa.^[65]^ After bradykinin is produced, it acts mainly on 2 receptor subtypes, B1 and B2. The B1 receptor mainly mediates the pain-causing effect of kinin,^[66]^ while the B2 receptor mainly mediates vasodilation.^[67]^ Current research suggests that bradykinin acts through G-protein-coupled receptors.^[68]^ Bradykinin binds to the B2 receptor to stimulate the release of nitric oxide, prostaglandin I2, and endothelium-dependent hyperpolarizing factor, resulting in a strong vasodilation effect.^[69]^ Although bradykinin’s vasodilatory effect benefits the heart, its accumulation also leads to angioedema.^[70]^ Because neprilysin inhibition involves variable peptides and many pathways, the precise mechanisms of the ventricular arrhythmia reduction effects of neprilysin inhibition are complex and poorly defined. Further research is required to elucidate this mechanism.

**Figure 3.**
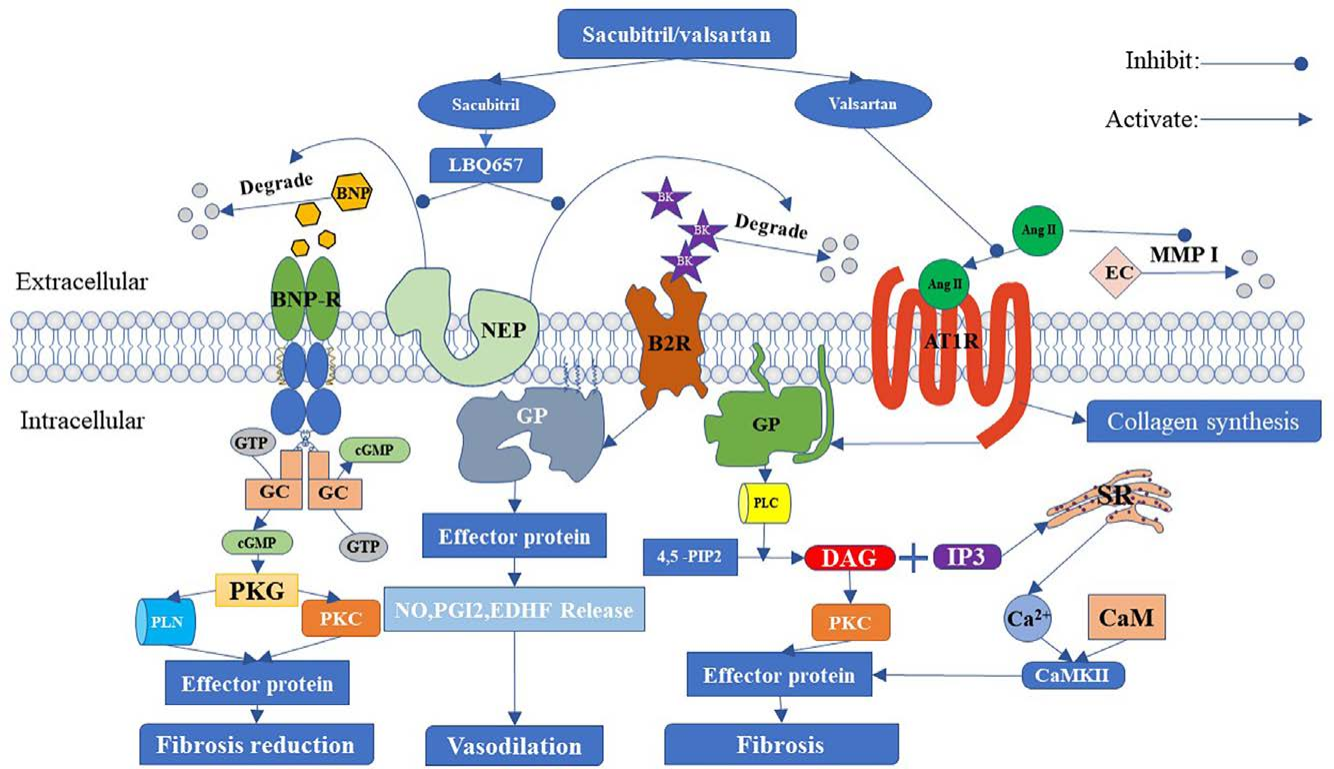
Possible mechanisms underlying the antiarrhythmic effect of sacubitril/valsartan. 4,5-PIP2 = 4,5-bisphosphate phosphatidylinositol, Ang II = angiotensin II, AT1R = angiotensin type 1 receptor, BNP = B-type natriuretic peptide, B2R = B2 receptor, BK = bradykinin, BNP-*R* = B-type natriuretic peptide receptor, CaM = calmodulin, CaMKII = calmodulin-dependent protein kinase II, cGMP = cyclic guanosine monophosphate, DAG = diacylglycerol, EC = extracellular collagen, EDHF = endothelium-dependent hyperpolarizing factor, GC = guanylate cyclase, GP = G protein, GTP = guanosine triphosphate, IP3 = triphosphate, MMP I = matrix metalloproteinase I, NEP = neprilysin, NO = nitric oxide, PKC = protein kinase C, PGI2 = prostaglandin I2, PKG = protein kinase G, PLC = phospholipase C, PLN = phospholamban, SR = sarcoplasmic reticulum.

In general, sacubitril and valsartan may reduce the occurrence of myocardial fibrosis and ventricular arrhythmia through the BNP, angiotensin II, and bradykinin pathways. Among them, the result of acting on the BNP and angiotensin II pathways is fibrosis reduction, and the result of acting on the bradykinin pathway is vasodilation. Subsequently, vasodilation can eventually lead to a reduction in cardiac afterload and the occurrence of ventricular arrhythmias (Fig. 3).

## 5. Current problems associated with the clinical use of sacubitril/valsartan

Although the positive effects of sacubitril/valsartan in patients with heart failure have been widely confirmed, there are still some problems with its clinical application.

### 5.1. Symptomatic hypotension

In the PARADIGM-HF trial, the incidence of hypotension in the sacubitril/valsartan group was 14%, which was significantly higher than that in the enalapril group.^[3]^ Ferre-Valverdu et al^[10]^ studied 322 patients with HFrEF treated with sacubitril/valsartan. The results showed that 12.4% of the patients could not tolerate sacubitril/valsartan owing to the development of symptomatic hypotension, renal dysfunction, gastrointestinal symptoms, and hyperkalemia. Symptomatic hypotension is the main obstacle to achieving the necessary clinical experimental dose, followed by deterioration of renal function, hyperkalemia, and gastrointestinal symptoms. Hypotension that limits the use of heart failure drugs is a common problem in the elderly, and hypotension in the elderly is associated with worse outcomes in heart failure.^[71]^

### 5.2. The optimal dosage is not individualized

Hsu et al^[72]^ think that because Asian people often have a lower body mass index, lower blood pressure, and are more intolerant to the clinical dose of ACEIs/ARBs, this population may not be able to tolerate the dose of sacubitril/valsartan used in clinical trials. At present, the recommended dose given by European research results may not be suitable for Asian populations. A large-scale research is needed to determine the correct dosage of sacubitril/valsartan for Asian populations.

### 5.3. The problem of drug initiation

Patients with heart failure in the PARADIGM-HF trial were previously treated with ACEI or ARB, but the trial did not address the possibility of safe initiation of sacubitril/valsartan therapy in HFrEF patients who were not sensitive to RAAS inhibition.^[3]^

### 5.4. Sacubitril/valsartan may cause arrhythmia and advance the appearance of sudden cardiac death

Although sacubitril/valsartan can reduce ventricular arrhythmia, Vicent et al^[16,22]^ reported that 6 patients with heart failure developed ventricular arrhythmia after sacubitril/valsartan treatment, which disappeared after drug withdrawal. Similarly, a study by Gatti et al^[20]^ showed that sudden cardiac death occurred early after sacubitril/valsartan administration. These reports raise the question of whether sacubitril/valsartan has an arrhythmogenic effect. However, the patients included in the above studies were relatively ill, and many had ischemic heart disease. One previous study has suggested that changes in myocardial perfusion conditions caused by ischemia may also lead to arrhythmias.^[73]^ Therefore, more clinical studies are needed to confirm the validity of this finding.

### 5.5. Sacubitril/valsartan may increase the risk of Alzheimer disease

Long-term use of sacubitril/valsartan inhibits neprilysin, which plays an essential role in degrading the β-amyloid protein. Therefore, sacubitril/valsartan could result in amyloid deposition in the brain, raising concerns about whether the drug will increase the risk of Alzheimer disease. Clinical studies are underway to assess whether the drug affects cognitive function, and the results are expected to be published soon.^[74,75]^

### 5.6. Limitations of current research

Current research is limited to treating ventricular arrhythmias in patients with HFrEF, and the use of sacubitril/valsartan in patients with heart failure with preserved ejection fraction is still at the research stage. Therefore, the scope of the antiarrhythmic application of sacubitril/valsartan is currently limited, and more clinical trial results are needed to determine whether sacubitril/valsartan can be used to treat ventricular arrhythmia.

## 6. Conclusion and outlook

The clinical use of sacubitril/valsartan has dramatically improved the prognosis of patients with HFrEF and reduced the incidence of sudden death in patients with heart failure. Reduction in myocardial fibrosis may be one of the reasons for the reduction in ventricular arrhythmia. Furthermore, the reduction of ventricular arrhythmia caused by sacubitril/valsartan may be vital in reducing the rate of sudden death in patients with heart failure. Collectively, sacubitril/valsartan reduced the occurrence of ventricular arrhythmia in multiple ways. However, further studies are needed to elucidate the complex mechanisms by which sacubitril/valsartan changes the electrophysiology of the heart. At the same time, the potential proarrhythmic effects of sacubitril/valsartan reported in some studies need to be confirmed in more studies.

## Author contributions

Z.W. conceived, wrote, and revised the manuscript. M.Z., Q.Z., L.G., X.W., and Z.W. participated in the discussion and provided suggestions. M.G. provided financial support for writing the manuscript and the proposed amendments. Z.Z. provided guidance for writing the manuscript. All authors have read and approved the final manuscript.
